# A Thesaurus for Soil Invertebrate Trait-Based Approaches

**DOI:** 10.1371/journal.pone.0108985

**Published:** 2014-10-13

**Authors:** Benjamin Pey, Marie-Angélique Laporte, Johanne Nahmani, Apolline Auclerc, Yvan Capowiez, Gaël Caro, Daniel Cluzeau, Jérôme Cortet, Thibaud Decaëns, Florence Dubs, Sophie Joimel, Muriel Guernion, Charlène Briard, Fabien Grumiaux, Baptiste Laporte, Alain Pasquet, Céline Pelosi, Céline Pernin, Jean-François Ponge, Sandrine Salmon, Lucia Santorufo, Mickaël Hedde

**Affiliations:** 1 INRA, UR251 « PESSAC », Versailles, France; 2 CESAB/FRB, Aix-en-Provence, France; 3 German Centre for Integrative Biodiversity Research (iDiv), Leipzig, Germany; 4 Bioversity International, Montpellier, France; 5 Centre d’Ecologie Fonctionnelle et Evolutive (CEFE), CNRS, Université of Montpellier II, Montpellier, France; 6 Laboratoire Sols et Environnement, UMR 1120 INRA, Vandoeuvre-lès-Nancy, France; 7 INRA, UR1115 « Plantes et Systèmes Horticoles », Avignon, France; 8 Université de Rennes 1, UMR CNRS 6553 « EcoBio », Paimpont, France; 9 Université Paul Valéry Montpellier III, Centre d’Ecologie Fonctionnelle et Evolutive, Laboratoire de Zoogéographie, UMR 5175 CEFE, Montpellier, France; 10 UFR Sciences et Techniques, EA 1293 « ECODIV », Université de Rouen, Mont Saint Aignan, France; 11 IRD, UMR IEES-P, Bondy, France; 12 Université de Lille 1, EA 4515 « Laboratoire Génie Civil & géo Environnement », Lille Nord de France, Ecologie Numérique et Ecotoxicologie, Villeneuve d’Ascq, France; 13 Université Lille Nord de France, Ecole Supérieure du Professorat et de l’Education (ESPE), Arras, France; 14 UR AFPA, Faculté des Sciences et Technologies, Université de Lorraine, Vandœuvre-lès-Nancy, France; 15 CNRS, UMR 7179, Muséum National d’Histoire Naturelle, Brunoy, France; 16 Department of Structural and Functional Biology, University of Naples Federico II, Naples, Italy; Rockefeller University, United States of America

## Abstract

Soil invertebrates are known to be much involved in soil behaviour and therefore in the provision of ecosystem services. Functional trait-based approaches are methodologies which can be used to understand soil invertebrates’ responses to their environment. They (i) improve the predictions and (ii) are less dependent on space and time. The way traits have been used recently has led to misunderstandings in the integration and interpretation of data. Trait semantics are especially concerned. The aim of this paper is to propose a thesaurus for soil invertebrate trait-based approaches. T-SITA, an Internet platform, is the first initiative to deal with the semantics of traits and ecological preferences for soil invertebrates. It reflects the agreement of a scientific expert community to fix semantic properties (*e.g*. definition) of approximately 100 traits and ecological preferences. In addition, T-SITA has been successfully linked with a fully operational database of soil invertebrate traits. Such a link enhances data integration and improves the scientific integrity of data.

## The Need for Semantic Data Integration for Soil Invertebrate Traits

The soil fauna consists of a variety of animals which may represent as much as a quarter of all currently described biodiversity [1]. Of this, soil invertebrates are known to be highly involved in soil behaviour (*e.g.* carbon transformation and sequestration, soil aggregation) and therefore in the provision of ecosystem services [2]–[4]. As a consequence, soil ecologists aim to understand the interactions between soil invertebrates and their environment. Functional trait-based approaches are methodologies which can help us to understand soil invertebrates’ response to their environment through their traits. In this paper, we consider functional traits as being characteristics of individuals that affect their fitness and govern their responses to their surrounding environment [5]–[8]. The main advantages of trait-based approaches are that they (i) improve the prediction of the relationship between soil invertebrates and environmental changes and (ii) reduce the dependence of such predictions on time and space [8]. Trait-based approaches have confirmed the existence of environmental filters which filter a sub-set of individuals from the regional pool to form local communities [9]. Furthermore, trait-based approaches have been shown to be reliable over eco-regions and for whatever kind of environmental change is considered [10].

The current use of traits for soil invertebrates resulted from isolated initiatives which produced large amounts of unconnected heterogeneous data [8]. Without efforts to integrate such data, the emergence of new knowledge from combining, reusing or sharing it will remain scarce and time-consuming. Our main aim is to provide soil invertebrate scientists with tools which allow data identification, availability and interoperability [11]. The semantic web offers such kinds of tools by being based on the key principles of metadata, controlled vocabularies and ontologies [11].

The integration of the trait data on soil invertebrates is a key issue which can be resolved through semantic data integration. It deals with the variation of the terms employed (terminology) over time. It preserves their meanings (scientific concepts) and also captures their interrelationships [12], [13]. In the following, trait scientific concepts will be called “concepts” and trait terms which pertain to trait scientific concepts will be called “terms”. As has been stressed by some authors, semantic inconsistencies can not only impede data integration [14] but could also lead to ambiguous scientific data interpretation [13]. For instance, concerning the problem of data integration, some authors employed either the term “body length” [15] or “body size” [16] to describe the same concept, *i.e.* the length of the body, for two soil invertebrate taxonomic groups respectively. Without a semantic link between these two terms, data integration is impossible as data were described by two different terms. Is the concept associated with these two terms the same? Only a semantic structure would remove any doubt and identify these two terms as synonyms. Such semantic inconsistencies also exist within a given soil invertebrate taxonomic group. For instance, the development of ground beetle wings has been called “wing morphology” [17], [18], “wing form” [10] or “wing type” [19]. Otherwise, concerning examples of misunderstanding scientific data interpretation, the type of food eaten by soil invertebrates (*e.g.* carnivorous which means that they eat animals, usually alive), the way they feed on them (*e.g.* predators, which means that they feed by killing their live prey) or finally their trophic position in the food chain (*e.g.* tertiary consumers which eat animals feeding on herbivores) refer to different concepts. Nevertheless, the literature contained several categorical traits whose attributes described several of the above concepts simultaneously. For instance, the term “food of the adult” [20], [21] referred both to the type of materials ingested (*e.g.* plants, springtails) but also to the way the materials were eaten (*e.g.* generalist predators). The terms “feeding guilds” [22] or “food strategy” [23] are other terms used. Another example is that, to refer to the body colour, some authors employed the term “body colour” for carabid beetles [21], [24] while others used “body pigmentation” for earthworms [15], [25]. However, the concept of “coloration” is different from the concept of “pigmentation” since pigmentation does not necessarily imply the presence of colour. As soon as the traits are not clearly defined, confusion will emerge from comparisons between trait data from multiple literature sources.

As far as we know there has been no attempt to deal with these shortcomings for soil invertebrates. One solution is to build a thesaurus, which is a list of terms used in a particular topic, with some of their properties, organized into a hierarchy according to their meanings, i.e. their concepts. The aim of this paper is to present a first thesaurus for soil invertebrate trait-based approaches, called T-SITA.

## The Thesaurus Construction

The thesaurus for soil invertebrate trait-based approaches (T-SITA) was constructed through a web-based tool, designed for the collaborative construction of thesauri in ecology: the Thesauform [13].

### 1. The tool: Thesauform features

The Thesauform allows a thesaurus to be created which resulted in a hierarchy of terms organized according to their meaning, i.e. their concept. Each term of the hierarchy is described by a defined number of its properties: preferred label, definition, bibliographic reference of the definition, abbreviation, synonym(s), related term(s) and preferred unit. The building procedure is performed collaboratively by a scientific expert community. It is divided into three successive steps: editing, validation and supervision. The editing step consists of the opportunity for each scientific expert to (i) modify and enrich the properties of a term, (ii) modify the hierarchy, (iii) add or delete a term or (iv) add a comment. The validation phase consists of a voting procedure within the scientific expert community on the different amendments produced during the editing phase. At each of these first two steps, several scientific experts can access the Thesauform simultaneously. The supervision phase aims to control the semantic consistency of the votes. It is mainly done by the editors of the thesaurus before the release of the final version. The whole procedure described above can be repeated indefinitely to continually improve the semantic content of the thesaurus.

### 2. The thesaurus for soil invertebrate traits: method

Before starting the editing phase, twenty-one experts in soil invertebrate ecology provided a list of approximately 80 well-known terms of traits and ecological preferences (see definitions in [8]). These trait/ecological preference terms were first chosen because they are commonly used for at least four notable invertebrate taxonomic groups with different biological strategies: earthworms, ground beetles, spiders and springtails. Nevertheless, the thesaurus design is not limited to such soil invertebrate groups. It is possible to input trait terms for all soil invertebrates and/or specific trait terms for a single soil invertebrate group (*e.g.* collembolan ocelli number).

**Figure 1:**
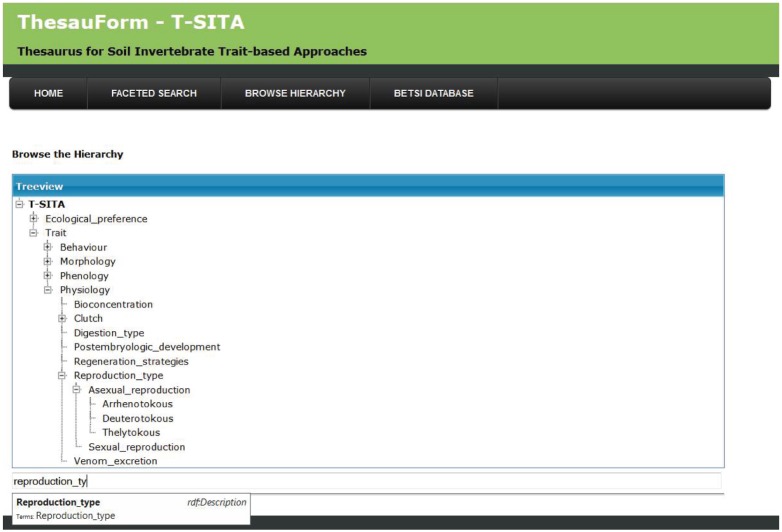
Auto-completed field and navigation tree searches in T-SITA.

Some of the properties of these selected trait and ecological preference terms (*e.g.* definition, unit, preferred label) were given as an input to the Thesauform system. They were organized in a conceptual hierarchical tree with their mother and daughter terms. Each term is conceptually included in its mother term. For instance, the “Reproduction type” trait term was included in the mother term “Physiology”. This means that the concept linked to the “Reproduction type” term is included in the concept linked to the “Physiology” term (Fig. 1). This last term is also included in the term: “Trait”. All the terms which have a position above a trait/ecological preference term, in the conceptual hierarchy tree, are called “multi-level mothers” of the trait/ecological preference term. For instance, the multi-level mothers of “Reproduction type” are: “Physiology” and “Trait” (Fig. 1). “Reproduction type” has two daughter terms: “Asexual reproduction” and “Sexual reproduction” (Fig. 1). We call “multi-level daughters” of a trait/ecological preference term all the terms which have a position below it in the conceptual hierarchy tree. For instance, the multi-level daughters of “Reproduction type” are: “Asexual reproduction”, “Sexual reproduction”, “Arrhenotokous”, “Deuterotokous” and “Thelytokous” (Fig. 1).

T-SITA has been coupled with a soil invertebrate trait database to improve data management and enrich trait/ecological preference information (see Section 4). To achieve this, a necessary condition is that each term must be typified by a unit. Trait and ecological preference terms are identified by having either a numerical unit or by being “categorical”. Quantitative traits require numerical values and therefore have numerical units. For instance, the unit of the “body length” trait term is mm. Otherwise, qualitative traits are described by textual data. To be usable, they need to be categorized into attributes, *e.g.* by fuzzy coding procedures [8], [26], so their units are described as being “categorical”. For instance, the unit of the “habitat” preference term is “categorical”. “Habitat” is categorized into several attributes, such as “agricultural area” or “wetland”. Such attributes appear as daughter terms of the habitat preference in the thesaurus hierarchy. Attributes are identified in the thesaurus by having a unit specified as being an attribute. For instance, the unit of the “Wetland” term is “attribute”. In addition, a categorical trait or ecological preference can have multi-level daughters. For instance, the habitat preference is the mother of “Agricultural area” which is itself the mother of “Arable land”, “Fallow” and “Perennial crop”. This was done to take into account the variable accuracy of the textual literature describing the categorical traits or ecological preferences.

To deal with a trait which is described by using a different data format, for instance from one soil invertebrate taxonomic group to the other, two different traits must be created. They must have the same definition but with different terms and units. For instance, there are in the thesaurus an “Antenna length” term (unit: mm) which represents an antenna length trait described by numerical data and an “Antenna categorical length” term (unit: categorical) which represents an antenna length trait described by textual data. Finally multi-level mothers of traits/ecological preferences have an empty unit. For instance, “Nutrition”, which is the mother of the “Mouthpart type” trait, has an empty unit.

The initial hierarchy was then inserted into the Thesauform and was made available to experts on the web at a URL address (no longer available).

From October 2011 to October 2012, experts carried out the editing phase. From October 2012 to April 2013, they carried out the validation phase. From April 2013 to October 2013, editors of the thesaurus checked the consistency of the thesaurus before its first available on-line version, as presented in this paper.

## The Thesaurus Content and Browsing

T-SITA is freely available at the following URL address: http://t-sita.cesab.org/Thesauform/BETSI_vizIndex.jsp. It contains 71 traits and 24 ecological preferences.

**Figure 2:**
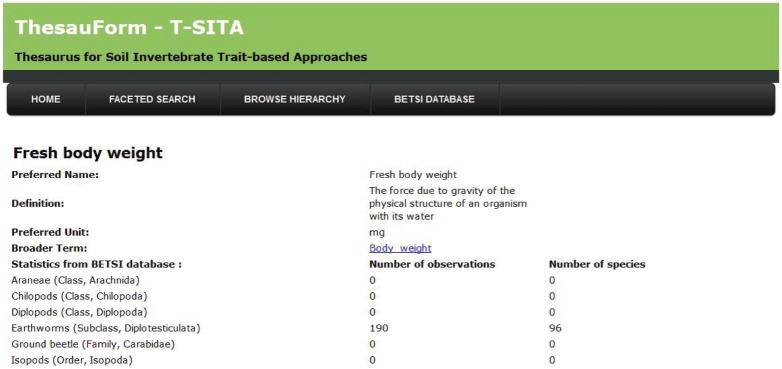
Panel of properties of the “fresh body weight” trait term in T-SITA.

Two types of semantic search engines are offered to access the T-SITA content. The first one is a classic semantic search engine which allows thesaurus terms to be found through an auto-completed search field and/or through a navigation tree (Fig. 1). It reflects how the experts of soil invertebrate ecology first organized the terms into a conceptual hierarchical tree and then amended it during the editing phase. To have access to information on a given term, it is necessary to click on it in the tree. Then a new web page appears with the properties of the term (Fig. 2).

**Figure 3:**
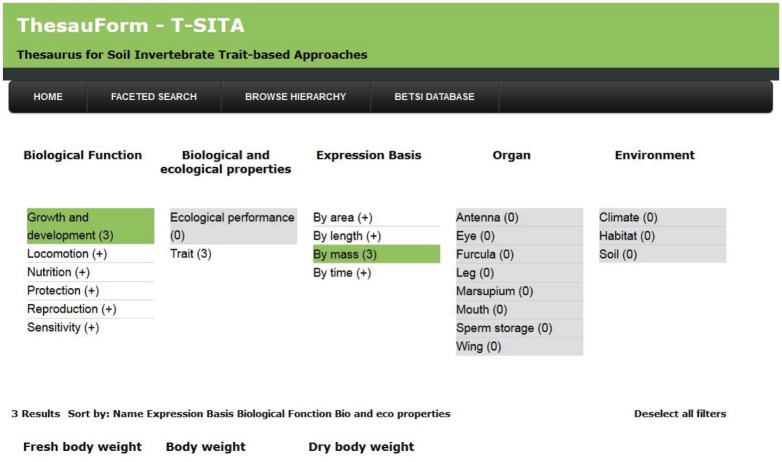
Faceted search system implemented to access T-SITA.

The second search engine available is a faceted search engine (Fig. 3). It is defined as being “a technique for accessing a collection of information allowing users to explore by filtering available information. It allows the assignment of multiple classifications to an object” [27]. It enables users to filter thesaurus terms on characteristics they share that are called ‘facets’. At the moment in T-SITA, terms can be filtered according to several facets which gather terms either by their expression basis, the organ concerned, the main biological function concerned, their nature by distinguishing the traits from ecological preferences [8] and finally by the environmental property concerned. Each facet is divided into several categories that the user can select (when selected, the categories are coloured green). For instance, the “expression basis” was divided into four categories (area, length, mass, time) so that the user can select one/several of them. A dynamically updated list of terms appears then in the result part according to the selected category(ies) (Fig. 3). Simultaneously multiple selection of facet categories is possible. For instance, the user can select simultaneously the category “growth and development” from the “biological function” facet and the category “by mass” from the “expression basis” facet. He will find three terms: the “body weight”, the “fresh body weight” and the “dry body weight” in the result part since they correspond to both filters (Fig. 3). To access the information on a given term, the user has to click on it in the result part. Then a new web page appears displaying the complete information on the term.

## A Useful Tool for Data Integration, Data Scientific Integrity and Navigation among Multiple Data Sources

The web of data focuses on the sharing of data on the web. SKOS (Simple Knowledge Organization System) (Isaac 2008) is the W3C standard dedicated to thesaurus representation, based on RDF triples [28]. SKOS was designed to provide an existing thesaurus standard (ANSI 2005) by providing a common format to adequately manage not only thesauri, but also any knowledge organizational system. T-SITA, built using Thesauform [13], is defined through the SKOS format and makes full use of it. As a consequence, T-SITA serves as a stable reference resource, specifically when available as linked data on the web. Additionally, T-SITA has been coupled with a soil invertebrate trait database to enhance data integration and data scientific integrity. The BETSI database will be soon in production. It is a relational database defined under the database management system PostgreSQL (http://www.postgresql.org/docs/9.1/static/reference.html), which contains soil invertebrate data on traits and ecological preferences. The linkage of the thesaurus and the database presents huge advantages.

First, each insertion of trait data in the BETSI database is under the control of T-SITA, which improves data quality in the database. Indeed, a trait term must be present in T-SITA in order to insert related data in the BETSI database, so data contributors to the BETSI database must consult T-SITA before inputting data. It guides the data integration and limits scientific misunderstanding. Concerning the data integration, it allows integrating trait data from two trait terms that represent the same concept (see section 1). For instance, sclerotization of the body is called “integument sclerotization” or “cuticule sclerotization” in the literature. As both terms are defined as synonyms in T-SITA, data inserted from these two terms will be identified in the database as belonging to the same trait concept. As a consequence, a data query concerning the sclerotization of the body will return data from both terms. Concerning the data interpretation, contributors to the database have to associate their data with a term coming from a limited set of trait terms. Each of them represents a trait concept which does not overlap with other trait concepts in the thesaurus. Two trait terms representing the same concept are synonyms. This can resolve the problems of scientific misunderstanding mentioned in section 1. Data contributors who want to insert raw data describing the “body colour” trait for carabid beetles and the “body pigmentation” trait for earthworms will realize that both data apply to the same concept. As a consequence, they will insert the data under the same trait term “body colour” in T-SITA and not under the trait term “body pigmentation” which refers to a different concept.

Second, in the web page describing the trait/ecological preference information in T-SITA, the dynamic link with the database enriches information about a given trait/ecological preference by providing statistics coming from the BETSI database (Fig. 2). It displays for each trait or ecological preference (as soon as a unit has been allocated to them, see section 2), the number of raw data observations input into the database and how many species they concern (Fig. 2). In addition, the statistics are aggregated according to the level of the tree hierarchy. When clicking on an above term (no unit, see Section 2), the number of raw data observations input into the database and how many species they concern are aggregated from its trait/ecological preference daughter terms. For instance, the “body dimension” term displays the aggregated statistics coming from its daughter terms, i.e. the “body length” and “body width” trait terms.

Interoperability between T-SITA and the database is dynamic. Therefore, each time the content is modified either via the database or the Thesauform, the modification is instantaneously updated in the other tool without any human intervention.

## Conclusion

Harmonization of trait data for soil invertebrates requires a handbook to answer questions such as: what is really to be understood by this trait term, how can I measure it? T-SITA forms the first step in this huge task by being, to our knowledge, the first initiative to deal with the semantics of traits and ecological preferences for soil invertebrates. It reflects the agreement of a scientific expert community to fix the semantic properties (*e.g*. definition) of approximately 100 traits and ecological preferences. In addition, T-SITA has been successfully linked with a fully operational database on soil invertebrate traits. Such a link enhances data integration and improves data scientific integrity.

The future of T-SITA depends on the tool used to build it (Thesauform), which allows improvements in the current content by (i) performing other complete procedures (edit, validate, supervise), (ii) including other scientific experts and (iii) including new trait/ecological preference properties such as methods of measurement. Finally, a more long-term prospect for T-SITA could be its use for the construction of a soil invertebrate trait ontology.
